# Supplementary material for: Explaining the difference between men’s and women’s football
Source: PLoS One. 2021 Aug 4;16(8):e0255407. doi: 10.1371/journal.pone.0255407 (PMC8336886; doi:10.1371/journal.pone.0255407)
Supplement: S1 File — (PDF) [file pone.0255407.s001.pdf]

Explaining the difference between men's and women's football - Supplementary Information

July 20, 2021

Supplementary Information 1: Intensity of Play

We split a match into possession phases, i.e., sequence of consecutive events in which one team only owns the ball [1]. An action begins when a team gains the ball and ends if one of these cases occurs: the first half or the second half of match end, the ball goes out of the field, there is an offside or a foul [1]. In women's matches there is an event that is not present in men's matches, the so-called *cooling breaks*, i.e., pauses in the game due to excessive heat; the algorithm recognizes them and indicates them as an additional cause of end of action.

Average pass velocity. The average pass velocity $\text{PassV}(g)$ in a match g is the average time between two consecutive passes in which the receiver of the first pass is the player who makes the next pass to a teammate.

Average ball possession recovery time. The average ball recovery time $\text{RecT}(g)$ is the average time elapsed between a team's last recorded pass and the first new pass made by a player of the same team.

Shooting time. The average shooting time $\text{ShotV}(g)$ is the average time between two shots of the same team. For example, in the men's World Cup final, on average, for France approximately 345 seconds passed, and for Croatia about 281 seconds.

Average pass length. We measure the average pass length $\text{PassL}(g)$ in a match g as the average Euclidean distance between a pass's starting and ending positions.

Supplementary Information 2: PlayeRank scores

The PlayeRank algorithm takes into account different types of events made by the players to compute the performance rating $r(u, g)$ of each player u in a match g [2]. Given a match

g, PlayeRank describes the performance of a player u in g by a n-dimensional feature vector $Q_u^g = [x_1, ..., x_p]$, where each x_j, with $j = 1, ..., p$, is a feature describing a certain aspect of u's behaviour during g. Some features are related to the number of specific events produced by u in g (e.g., passes, shots), others take into account the outcome of these events, e.g., whether or not they are accurate. The performance rating $r(u, g)$ of u in g is computed as:

$$r(u, g) = \frac{1}{R} \sum_{i=1}^{p} w_j x_j \quad (1)$$

where w_j is the importance of feature j, x_j the value of that feature, and R a normalization constant. The weights w_j are computed during a learning phase based on machine learning and consisting of two steps: feature weighting and role detector training [2]. Note that PlayeRank assign every player to a role if they played at least 40% of the matches in that role. Each role in the field is defined through a K-means clustering method implemented in the role detection phase of the learning phase [2]. The performance rating $r(u, g)$ is combined with the number of goals scored using a goal weight α (set to $\alpha = 0.10$ in our experiments). For example, Harry Kane (England), in the match against Panama, scored three goals and achieved a PlayeRank score of 0.59, demonstrating its centrality in the 6 to 1 victory. Similarly, the Australian champion Samantha Kerr, in the match against Jamaica, scored four times resulting in a PlayeRank score of 0.80.

Supplementary Information 3: Team Indicators

H-indicator. The H indicator summarizes different aspects of the passing behaviour of a team T into a single value. All these aspects are related to the pass-based performance features, which are measured using a team's passing network in a certain match g. First, we compute the average amount μ_p of passes managed by players in a team during a match and the standard deviation σ_p of the amount of passes managed by players in a team during a match [3]. The higher σ_p, the higher is the heterogeneity in the volume of passes managed by the players. Moreover, we consider the distribution of passes over the zones of the pitch by splitting the football pitch into 100 zones, each of size 11 mt x 6.5 mt and computing the zone passing network, where nodes are zones of the pitch and edges represent the passes between two zones [3]. We take the average amount μ_z of passes managed by zones of the pitch during the match and the standard deviation σ_z of the amount of passes managed by zones of the pitch during the match [3]. High values of σ_z underlies the coexistence of hot zones with high passing activity and cold zones with low pass activity during the game. Low values of σ_z indicates, however, a more uniform distribution of the pass in game activity across the zones of the pitch [3]. Finally, we combine these indicators by

their harmonic mean to summarize the passing behavior of a team T into the H indicator:

$$H(T,g) = \frac{5}{(1/w + 1/\mu_p + 1/\sigma_p + 1/\mu_z + 1/\sigma_z)} \quad (2)$$

where w is simply the number of passes produced by the team T in a match g.

Flow Centrality. The team passing network allows measuring the centrality of each player within the network of passes. The team flow centrality derives from the player flow centrality [4], which we compute (and modify as needed) using the algorithm taken from [1]. The player flow centrality ranks each player based on their centrality in the network of passes in a certain match. Formally speaking, it measures the current-flow-betweenness-centrality value for each node (remembering that each node is a football player). The betweenness centrality captures a node's role in allowing information to pass from one part of the network to the other. Technically, it measures the percentage of shortest paths that must go through the specific node. The important thing to know is that betweenness is a measure of how important the node is to the flow of information through a network [5]. In this context, it quantifies how central a player is in passing the ball from one side of the field to the other. The team flow centrality is then defined by setting on average the betweenness flow centrality values of players of the same team T in the matches they played, $\mathrm{FC}_{avg}(T,g)$. We also compute a function to measure the variability $\mathrm{FC}_{std}(T,g)$ in the passing flow centrality of a team in a match. High values of $\mathrm{FC}_{std}(T,g)$ highlight that there are players that individually are at the center of a team passing behavior in a particular game g; low values of $\mathrm{FC}_{std}(T,g)$, otherwise, depict an equilibrium between players of the same team in the flow passing centrality.

Supplementary Figure 1 shows two examples of passing networks and the corresponding H, FC, and PR values.

Supplementary References

[1] Pappalardo, L. *et al.* A public data set of spatio-temporal match events in soccer competitions. *Scientific data* **6**, 1–15 (2019).

[2] Pappalardo, L. *et al.* Playerank: Data-driven performance evaluation and player ranking in soccer via a machine learning approach. *ACM Transactions on Intelligent Systems and Technology (TIST)* **10** (2019). URL `https://doi.org/10.1145/3343172`.

[3] Cintia, P., Giannotti, F., Pappalardo, L., Pedreschi, D. & Malvaldi, M. The harsh rule of the goals: Data-driven performance indicators for football teams. In *2015 IEEE International Conference on Data Science and Advanced Analytics (DSAA)*, 1–10 (2015).

[4] Duch, J., Waitzman, J. S. & Amaral, L. A. N. Quantifying the performance of individual players in a team activity. *PloS one* **5** (2010).

[5] Golbeck, J. *Introduction to Social Media Investigation: A Hands-on Approach* (Syngress, 2015), first edn.

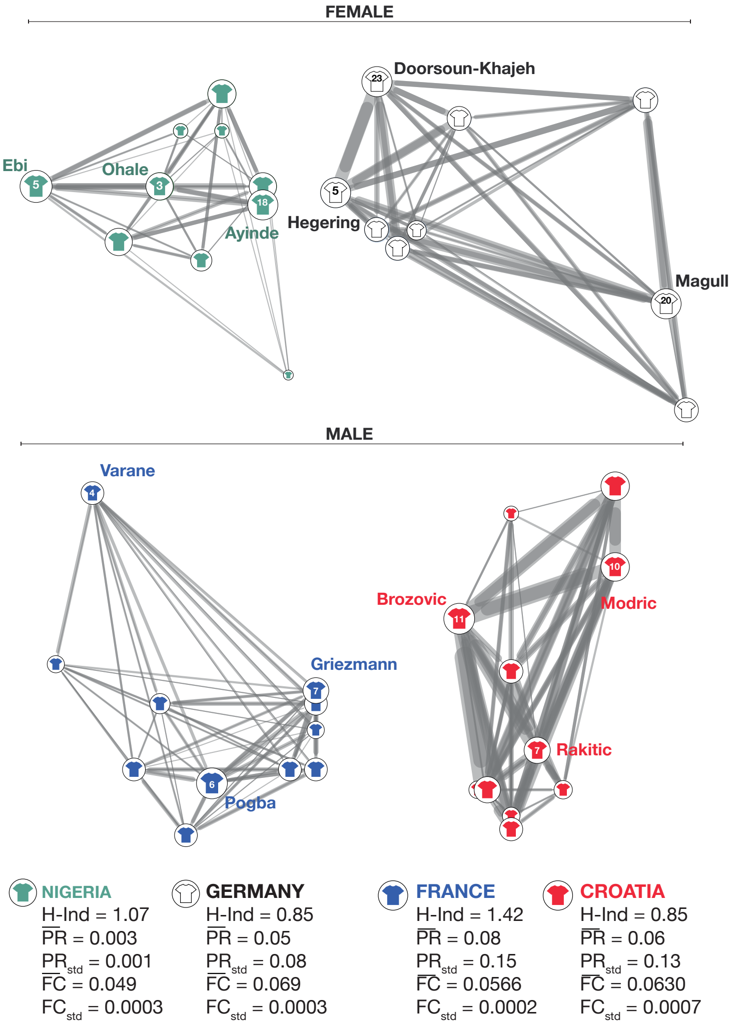

Supplementary Figure 1: Passing network of the France World Cup game of the *round of 16*, Germany v. Nigeria, and the Russia World Cup *final*, France v. Croatia. Each node represents a player and its width is related to how many times teammates have passed the ball to that particular player. Formally, the width is related to the normalized weighted in-degree measure. The edges width, however, is weighted with respect to how many times two players have passed the ball to each other. There are highlighted the players who received the highest percentage of passes from their team mates, i.e., the most sought after on the pitch during the match. The algorithm used to draw the network was taken and modified as needed from the article [1].
