# Supplementary material for: Explaining the difference between men’s and women’s football
Source: PLoS One. 2021 Aug 4;16(8):e0255407. doi: 10.1371/journal.pone.0255407 (PMC8336886; doi:10.1371/journal.pone.0255407)
Supplement: Attachment — Submitted filename: Revision R1 .pdf [file pone.0255407.s002.pdf]

# Summary of changes for revision

May 2021

## Explaining the difference between men's and women's football

Luca Pappalardo, Alessio Rossi, Michela Natilli, Paolo Cintia

We thank the Referees for their insightful comments. In particular, we are glad that Referee 1 says: “I found this to be a very innovative and informative paper” and “findings are descriptively strong and make considerable (intuitive) sense and therefore help to build a foundation for a new line of research”, and that Referee 2 says “I find the study interesting, especially the first part attempting to identify variables that differ between men’s and women’s football”.

We addressed all the Referee’s concerns in the new version of the manuscript. The parts that have been modified with respect to the previous version are highlighted in blue.

We believe that the manuscript has improved significantly thanks to the comments of the reviewers, and we hope that now the paper meets the high standards to be published in PLoS One.

Kind Regards, on behalf of all authors
Luca Pappalardo

# Reviewer #1

## Overall comment by the Referee:

*I found this to be a very innovative and informative paper. As the authors note, comparisons of male and female soccer have only recently received empirical attention, and this study provides an informative overview of major differences in technical play, with a focus on spatio-temporal events, along with individual and collective performance. Findings are descriptively strong and make considerable (intuitive) sense and therefore help to build a foundation for a new line of research. I must admit that I do not have the expertise to evaluate the statistical analyses, so I hope that this is covered by other reviewers. But focusing on my expertise and what I can evaluate, I believe this paper is a very strong one.*

## Response

We thank the Referee for the appreciation of our paper and the very positive comments, which helped clarify important aspects of our work.

## Point 1.1

*I found the paper a real pleasure to read. At the same time, the paper's readability can be improved if the authors use labels that help readers to immediately grasp the meaning. For example, the indices of H, PR, and FC are not linked to any meaning, it seems. Why not use meaningful labels, which the authors do for FC (Flow centrality). Also, H and FC are two indicators of "collective" performance. It was not clear to me whether they were correlated. For example, when I look at Table 3, it seems that H and FC are not strongly correlated, because there are not so many overlapping countries in the top 10.*

## Response 1.1

We thank the Referee for this useful comment, which gives us the opportunity to clarify the difference between the considered performance metrics.

H, PR and FC are three metrics that describe the teams' performance quality. In particular, they refer to the H-indicator[1], the PlayeRank score[2] and the Flow Centrality[3], respectively. These metrics, that we describe in detail in the Supplementary Material, evaluate different aspects of a team's performance. No significant correlation was detected among these

---

[1] Cintia, Paolo, et al. "The harsh rule of the goals: Data-driven performance indicators for football teams." 2015 IEEE International Conference on Data Science and Advanced Analytics (DSAA). IEEE, 2015.

[2] Pappalardo, Luca, et al. "PlayeRank: data-driven performance evaluation and player ranking in soccer via a machine learning approach." ACM Transactions on Intelligent Systems and Technology (TIST) 10.5 (2019): 1-27.

[3] Duch, Jordi, Joshua S. Waitzman, and Luís A. Nunes Amaral. "Quantifying the performance of individual players in a team activity." PloS one 5.6 (2010): e10937.

collective parameters (H vs PR: $r = -0.12$, $p < 0.01$; H vs FC: $r = 0.07$, $p < 0.01$; PR vs FC: $r = 0.32$, $p < 0.05$). Hence, these three features describe different aspects of the team performance.

The PR score (which stays for PlayeRank score) quantifies a player's performance quality in a match based on all the events type (e.g., pass, shot, and duel), resuming the players performance goodness by a data-driven approach. We then aggregated the PR score to obtain the average and variance of performance quality at the team level.

H and FC indices are both related to a team's passing network, but they capture different aspects of a team's performance. On the one hand, the H-indicator summarizes different aspects of a team's passing behaviour, such as the volume of passes and the predictability of a team's passes. Note that the H-indicator is the name used in the paper that proposed the metric, this is why we left it as is. On the other hand, FC summarizes the team's player centrality, computed as the average player centrality in the passing network. The weak correlation between the two metrics may be explained by the fact that they quantify different aspects of a team's passing behaviour, which is actually the reason why we selected them.

Moreover, as the reviewer asserted in their comment, the teams' performance metrics shown in Table 3 (H and FC) are not very relevant to detect the gender of the team. (see Figure 3 of the manuscript). In contrast, the PR score is a relevant variable to the model.

We clarified the difference between the proposed performance metrics in Section 3.3. of the manuscript.

## Point 1.2

*One sizeable differences between men and women is the # of fouls (and more free kicks among women than men). The authors do pay much attention to it, but this seems quite interesting. This allows for at least a bit more interpretation.*

## Response 1.2

Thank you for this comment. We pointed out these aspects in Section 3.1 and 3.4, and throughout the conclusions (Section 5). In particular, we linked the differences on the number of fouls, free kicks and offside to the shorter recovery time observed in women matches compared to men ones. Moreover, we have highlighted that the number of fouls are lower in women matches compared to men ones, depicting resulting in a more correct/loyal game by women.

## Point 1.3

*The conclusions are straightforward. But I wonder whether it is desirable to provide a bit more discussion to the major findings, linking to the broader literature. What comes to mind*

*is a body of literature examining the role differences in biological make-up between men and women or the role of "cognition" (e.g., executive functioning) that might be relevant to understanding performance in soccer (see research by Lot Verburgh et al., 2014, PlosOne).*

## Response 1.3

We thank the Referee for this useful suggestion. In the new version of the manuscript, we deepened the discussion of our results in Section 5 (Conclusion).

In particular, we highlighted that some of the teams' technical characteristics are related to anthropometric and biomechanical differences between men and women (e.g., shot and pass distance). In addition, differences in technical skills between men and women may be related to the lower number of training hours performed by women.

Previous studies [31, 32] demonstrate that training time is related to sporting level suggesting that a longer time spent on specific tasks is required to increase the soccer player technical capabilities [7]. Perroni et al. [7] suggest that increasing the training time of specific technical capabilities is crucial to make the training of women soccer players more effective. Additionally, due to the dynamic nature of soccer technical skills, individual technical capabilities should be trained incorporating neuromuscular (i.e., strength) and cognitive (i.e., decision-making and visual searching processes) functions in both women and men soccer players [7]. As a matter of fact, Verburgh et al. [33] demonstrate that talented players result in higher cognitive functions (i.e., motor responses and the ability to attain and maintain an alert state essential for success in soccer) and higher technical skills compared to amateur players. Although women's football's technical level is increasing rapidly, there is still a technical gap between the two sports.

We adapted the above discussion in Section 5 (conclusions) of the manuscript.

## Point 1.4

*The paper needs to be checked on typos (e.g., length rather than length, for rather than for, etc). Also, I noted that the Dutch name "Paul A.M. Van Lange" should be read as "Van Lange" (not "Lange") both in the text and references.*

## Response 1.4

We thank the Referee for noticing the typos. We carefully checked for all the typos and improved the writing of the paper.

# Reviewer #2

## Overall comment by the Referee

*The manuscript reports a study attempting to identify variables that can distinguish between men's and women's football (soccer). The aim was to train an AI-model so that it can recognize whether a team is male or female. The results reveal differences between men's and women's football on several variables and the model, based on computed performance indicators from selected variables, was able to correctly identify a team's sex in around nine out of ten cases.*

*I will not claim to be an expert on the specific methods used for building the model, so my comments are related to the choice of variables, the interpretation of the results, and the conclusions. In particular, I am concerned with the validity of the results, and consequently their usefulness.*

## Response

We would like to thank the Referee for their appreciation of our paper and for the useful comments, which helped improve the paper significantly.

## Point 2.1

*I find the study interesting, especially the first part attempting to identify variables that differ between men's and women's football. I would have liked to see more of this information in the paper and not only as supplementary information (for example the heatmaps showing areas where free-kicks and shots were taken), and I would have liked to see more discussion related to these differences and their possible consequences.*

## Response 2.1

Thanks for this useful comment, which allows us to improve the readability of the paper. We have moved and properly discussed two figures from the Supplementary Material to the manuscript: *(i)* the heatmaps describing the pitch zones from where the free-kick shots and the shots in motion are made in both women and men soccer matches (Figure 2); and *(ii)* the density plot of free-kicks and shots in the three pitch zones (i.e., Z1, Z2 and Z3) in both women and men soccer matches (Figure 3).

## Point 2.2

*According to the authors, "current studies focus on the physical features" (line 42), while their variables measure technical performance. However, some of the variables may be different between sexes due to other factors than technical abilities, and they may not be so*

*one-dimensional as they may seem. In fact, there are several possible confounding variables that could compromise the interpretation of the results.*

*I will give a few examples below:*

*• Several of the variables that are defined as technical, are in fact highly dependent on physiological factors. For example, passing length, as well as shooting distance require (leg-) muscle strength and are also dependent on biomechanical factors that are different between the sexes, notably torques. Thus, the differences may be rather obvious, and they may not reveal any important information about technical performance.*

*• There are trade-offs between tactical and technical variables such as for example pass length and pass accuracy. A team that plays longer passes may well use this as a strategy against teams that are more passing-oriented, or as a general strategy if the players are not so technically fluent. Also, a team that plays out from the back would generate plenty more passes, and also higher passing accuracy due to many passes being less risky, whereas a team that more often played out long from the goalkeeper would generate longer passes on average, and at the same time increase the risk, thus decrease the accuracy. Hence, the variable may be contaminated by differences in playing styles, regardless of sex.*

*• The average pass length, as well as the average shot distance, is not very much shorter in women (1 m, and 1.5 m, respectively) with considerable overlap as is evident from the standard deviations. This means that, although there is an average difference between the sexes, there are so large within-sex variations that the variable is rather poor at distinguishing between teams. For illustration, in the FIFA WC 2018, according to fbref.com the average shot distance varied between 22 m (Saudi Arabia) and 15 m (Serbia). 15 of the male teams had average shot distances below the female average (18.39 m, according to the present manuscript). In the Women's FIFA WC 2019, the average shot distance varied between 25 m (Argentina) and 15 m (the Champions, USA). Six female teams had average shot distances above the male average of 19.99 m.*

## Response 2.2

We agree with the Referee's comment. Some of the differences detected between the technical variables of women and men are linked to their physiological differences that could also affect the playing style. Hence, we stated in the paper that differences in technical and physical characteristics between women and men are associated with a difference in matches' events.

We have developed Section 5 (Conclusion) by pointing out the aspects raised by the reviewer in this comment.

## Point 2.3

*My biggest concern is with the validity of the results, thus what can be concluded from them, apart from the fact that it is possible, in most cases to identify a team by its sex. Exactly what*

*is the model identifying? I am not completely convinced that it is performance quality, and that it is performance quality that differs between sexes.*

## Response 2.3

As the Referee points out, it may be that the algorithm is detecting differences in performance among the teams. This is actually what we want to show: there are significant differences in the performance of men and women, to such an extent that a classifier is able to discriminate between men and women matches on the basis of these performance characteristics. We clarified this aspect throughout the new version of the manuscript and in particular in Section 4 (Team gender recognition) of the manuscript.

## Point 2.4

*The algorithm was generally able to categorize matches and teams by their sex (how were the matches selected; randomly?). However it made errors in around ten percent of cases.*

## Response 2.4

We split the dataset in two: we use 20% of the dataset to tune the models' hyper-parameters through a grid search with 5-folds cross validation; we use the remaining 80% of the dataset to test the model using a leave-one-team out cross-validation process. By this approach, we repeat the train and test split for *n* folds (*n* = number of teams in the dataset). For each fold, we train the model on a training set and test the model on a test set consisting of all the matches of a specific team (the one not covered in the training set). The models' accuracy refers to the average accuracy over all the folds.

AdaBoost is the model among the ones tried in our work, with an accuracy of 93%, considerably higher than the accuracy of a baseline model (48%). These results indicate that a classifier can accurately distinguish between male and female teams on the only basis of the performance variables. The 7% of error made by AdaBoost classifier is extremely low compared to the error of the baseline model (66%). Hence, only a few examples are misclassified by the model due to the fact that “extreme” (both positive and negative) performances in both male and female matches exist.

We have deeply described the validation approach and the model goodness in Section 4 (Team gender recognition), Moreover, in Figure 9 we provide two examples of match misclassification explaining the reason why some matches are wrongly classificated.

## Point 2.5

*A model is only as good as its variables, and variables that are used to conclude about differences in quality of performance, would need to be validated against actual performance, which is what we can deduct from Table 3. None of the three indicators seem*

*particularly sensitive to sex differences, with both male and female teams among the top scorers. I would have liked to see the complete rankings, which I suspect may lend some explanation to the fact that the model sometimes mischaracterizes teams as belonging to the opposite sex.*

# Response 2.5

As the reviewer asserted in their comment, the teams' performance indices shown in Table 3 (H, FC and PR) are low sensitive to gender difference. In particular, H and FC parameters are not important features for machine learning models to discriminate between men and women matches as shown in Figure 3. In contrast, the PR score is a relevant variable to the predictor.

We clarify the difference between the proposed performance metrics in the new version of the manuscript (i.e., Section 3.3), adding the complete rankings (H, FC and PR) in Table 3. Moreover, we highlight in the text that these three features describe different aspects of the team performance. As a matter of fact, no significant correlation was detected among these collective parameters (H vs PR: $r = -0.12$, $p < 0.01$; H vs FC: $r = 0.07$, $p < 0.01$; PR vs FC: $r = 0.32$, $p < 0.05$).

# Point 2.6

*The model may not recognize male or female teams, but instead teams playing with a certain style. Furthermore, that playing style, whether by a male or a female team, may not be a valid indicator of the quality of performance. For these reasons, I would urge the authors to be much more prudent in their interpretation of their results, and in particular, their conclusions.*

# Response 2.6

We thank the Referee for their useful comments. As you asserted, the model may learn to discriminate between two playing styles. However, the only performance metrics that are relevant to the classifier's classification is the PR score, which captures how good players were on average during a match rather than the team's playing style. In contrast, H indicator and FC, which capture some aspects of a team's playing style, are not very relevant to the classifier's decisions.

For this reason, our interpretation of the results was that the model captures the differences in the performance and the technical characteristics of the teams, rather than their playing style. Furthermore, some features, such as Recovery time and Stop time, could be used to infer the intensity of playing, thus highlighting differences eventually arising among different competitions.

We have edited Section 5 (conclusions) to point out this aspect.
